# Supplementary material for: Fosfomycin Resistance Evolutionary Pathways of Stenotrophomonas maltophilia in Different Growing Conditions
Source: Int J Mol Sci. 2022 Jan 20;23(3):1132. doi: 10.3390/ijms23031132 (PMC8835530; doi:10.3390/ijms23031132)
Supplement: Supplementary file 1 [file ijms-23-01132-s001.zip › ijms-1535333-supplementary.pdf]

Table S1. Primers used in this study

Primer	Sequence (5´- 3´)	Description
eno_SNP_F<br>eno_SNP_R	GCCTCGACCGTCG<br>CCGACTTCAGCGAATGG	Verification of Leu106Pro in *eno*
phaR_SNP_F<br>phaR_SNP_R	GACACCGAGATCTCCAGCTA<br>CCAGATAGTTGCCCATGAAG	Verification of Ser85Phe in *phaR*
bolA_SNP_F<br>bolA_SNP_R	CGGAAGTAGAATGGAGGCAC<br>GTTCACCCAGCAGCTTCAC	Verification of Ala52Ala in *bolA*
virB10_ins_F<br>virB10_ins_R	TTGGAGACATTTTCCACGTC<br>AGTAGGCCACAAGAAGATCG	Verification of Asp173fs in *virB10*
recR_ins_F<br>recR_ins_R	CCGAGTCGAAGTCGAAGAT<br>TCGATCAATTGTTCAAGCAG	Verification of CAAGCGGGTGCCACAGAA insertion
gspL_SNP_F<br>gspL_SNP_R	GTCGTGATGCTGGTACTGG<br>GGGTCAGTTCGTTCCAGATT	Verification of Val247Phe in *gspL*
ptsN_SNP_F<br>ptsN_SNP_R	GTCGTGATGCTGGTACTGG<br>GGGTCAGTTCGTTCCAGATT	Verification of GlnAlaLeu126ArgProAla in *ptsN*
SMD_RS14605_SNP_F<br>SMD_RS14605_SNP_R	CTTCGTGTTCGAGAACGTC<br>AGCTCCTCGATCACTTCCTC	Verification of Thr419Pro in SMD_RS14605
27<br>48	TGCCAGCGACAGTGCAAAGGGTC<br>CCGTGTTCATGGAAGCAGGC	Amplification of *smeT* to test DNA contamination
gyrA_F<br>gyrA_R	CGCAAGAGCTACCTCGATTA<br>GGTGGTACTTACCGATGACG	To amplify the housekeeping gene *gyrA* by real-time PCR
murA_F<br>murA_R	TGTCCAAGCTGGTCGAAG<br>TCGAAGATCGTTTCGTTGAT	To amplify *murA* by real-time PCR
bolA_F<br>bolA_R	ACACCATCCGCAACCTGAT<br>CAGGGTGGCATAGACCAT	To amplify *bolA* by real-time PCR
virB10_F<br>virB10_R	GTCCAAAATGTCTCCAATGC<br>ATAGATCTTGGAGCCCTTCG	To amplify *virB10* by real-time PCR
recR_F<br>recR_R	GAACAATTGATCGACGCACT<br>CTGCTGTTGGCACAGGTC	To amplify *recR* by real-time PCR

Primer	Sequence (5´- 3´)	Description
gspL_F<br>gspL_R	GTCGTGATGCTGGTACTGG<br>CAGTTCGTTCCAGATTTCAA	To amplify *gspL* by real-time PCR
SMD_RS14605_F<br>SMD_RS14605_R	GGTACGCTGGGCTACTACAC<br>TCTGGAAGAACGAGAACAGG	To amplify *SMD_RS14605* by real-time PCR
ptsN_F<br>ptsN_R	TGCTGGTGGGTGTAGTGG<br>GACAGATCTATCTGAACCTGTGC	To amplify *ptsN* by real-time PCR
